# Lysis of membrane lipids promoted by small organic molecules: Reactivity depends on structure but not lipophilicity

**DOI:** 10.1126/sciadv.aaz8598

**Published:** 2020-04-22

**Authors:** Hannah M. Britt, Aruna S. Prakash, Sanna Appleby, Jackie A. Mosely, John M. Sanderson

**Affiliations:** Chemistry Department, Durham University, Durham DH1 3LE, UK.

## Abstract

Several organic molecules of low molecular weight (<150 Da) are demonstrated to have substantial membrane-lytic potential despite having a low predicted lipophilicity (log*D* < 1 at neutral pH). In aqueous liposome dispersions, 38 aromatic compounds were tested for their ability to either promote lipid hydrolysis or directly participate in chemical reactions with lipid molecules. Behaviors observed included acyl transfer from the lipid to form a lipidated compound, both with and without concomitant lysolipid formation; increases in the rate of lipid hydrolysis without lipidation; and no reactivity. The variation in activity, including a notably higher activity for heterocycles such as amino-substituted benzimidazoles and indazoles, demonstrates the potential to predict or “design-in” lytic activity once the rules that govern reactivity are better understood. The nature of this chemical instability has significant ramifications for the use or presence of lipids in diverse fields such as materials chemistry, food chemistry, and cell physiology.

## Teaser

Organic molecules can induce the lysis of membrane lipids, either through direct reaction or by promoting lipid hydrolysis.

## INTRODUCTION

Direct chemical reactivity between lipids and the molecules that partition into membranes is very rarely considered. The chemical reactivity that is well described extends only to hydrolysis reactions involving bulk solvent (*1*–*3*) and autoxidation reactions (*4*–*6*), neither of which directly includes molecules that associate with membranes as part of their normal activity. In the eyes of many, lipid membranes, then, represent a stable environment into which molecules bind or insert as part of their normal function. In the classic work on the fluid-mosaic model, for example, it is stated that “…the phospholipids and proteins of membranes do not interact strongly; in fact, they appear to be largely independent” (*7*).

**Fig. 1:**
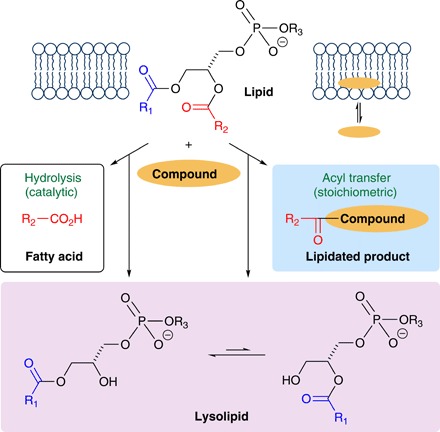
Lytic reactions of lipids in the presence of membrane-associated molecules. Reactions include acyl transfer from the lipid to the bound molecule and lipid hydrolysis catalyzed by the bound molecule. Both processes form lysolipids as coproducts. Note that, for clarity, only initial transfer of the *sn*-2 acyl group from the lipid is depicted, although the initial transfer of either acyl group is possible.

However, this notion of chemical stability has begun to be challenged. The transfer of a fatty acyl group from a membrane lipid to a membrane-embedded species (Fig. 1) has been described for peptides (aminolysis and transesterification) (*8*–*11*), and it has been suggested that similar reactions may be significant for some membrane proteins (*12*). Recently, similar acyl transfer reactivity was described for the drug propranolol, both in vitro and in vivo, to form a lipidated drug molecule (*13*). The reactivity of propranolol is of particular significance as it extends the observation of acyl transfer to low–molecular weight species, suggesting that membranes may be far more reactive than previously thought. It is currently still not clear how general this reactivity is, however, as other molecules, including fluoxetine, sertraline, chloroquine (*13*), haloperidol, spiperone (*14*), and raclopride (*15*), have been shown to promote the formation of lysolipids in model systems by hydrolysis (Fig. 1) without undergoing concurrent lipidation. Acyl transfer and hydrolysis may indeed be competing reactions, and the chemical landscape is further complicated by the potential for lipidated products to also catalyze lytic reactions of lipids, as well as the lysolipid products themselves, to be acyl donors in transfer reactions (while noting, in addition, that Fig. 1 shows a reaction of the acyl group from one site in the lipid when transfer of either is feasible). All the molecules described above that undergo lipidation reactions or influence the rate of lipid hydrolysis have a significant affinity for lipid membranes, and it is therefore not clear whether the reactivity will extend to lower–molecular weight species with much lower membrane affinity. Demonstration of such activity would not only change fundamental thinking on lipid membrane stability but also have additional implications for our understanding of membrane biology as there is some indication that lytic activity is responsible for downstream effects in vivo (*13*). Both the fatty acids and the lysolipids formed by these processes have substantial effects on membrane properties at abundance levels as low as 1 mol % due to their noncylindrical shape (*16*). Consequences of the presence of lipid lysis products include, for example, a notable increase in the permeability of the membrane and the ability to trigger lipid biosynthetic pathways (*17*).

**Fig. 2:**
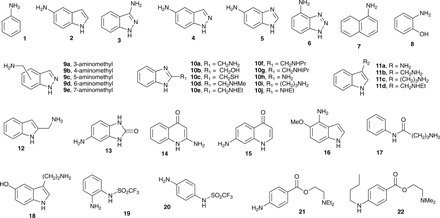
Compounds investigated in this study.

It is of fundamental interest to examine the extent to which organic molecules that partition reversibly into membranes are involved in lytic reactions. To that end, we examined a panel of compounds (Fig. 2) that include a range of aromatic and heteroaromatic ring systems combined with the presence of at least one nucleophilic center, covering a range (table S1) of predicted values for log*P* (−1.19 to 3.75), log*D* (−2.04 to 2.34), and p*K*_a_ (where *K*_a_ is the acid dissociation constant) (2.98 to 12.48) (*18*) for the nucleophilic center, as well as varying separation of the nucleophilic center from the ring. Our objectives were to establish that (i) low–molecular weight molecules with low predicted log*P*/log*D* can partake in membrane-lytic reactions (i.e., strong membrane interaction is not a requirement for lytic activity); (ii) reactivity exhibits selectivity according to molecular structure and, in principle, therefore can be predicted or designed; and (iii) the formation of lysolipids can be used as an overall measure of the lytic potential of a given compound.

## RESULTS

Each of the compounds in Fig. 2 was incubated under identical conditions with liposomes composed of 1,2-dioleoyl-*sn*-glycero-3-phosphocholine (DOPC) or a 4:1 mixture of DOPC with 1,2-dioleoyl-*sn*-glycero-3-phosphoserine (DOPS) and the concentration of the lysolipids 1-oleoyl-*sn*-glycero-3-phosphocholine (1-OPC) and 2-oleoyl-*sn*-glycero-3-phosphocholine (2-OPC) assayed by liquid chromatography–mass spectrometry (LC-MS) after periods of 24 and 72 hours. At the same time, analyses were conducted to directly detect the lipidated compound. Because of the unfeasibility of obtaining lipidated calibration standards for every compound tested, the results were interpreted qualitatively and are summarized in Table 1 and table S2.

**Table 1:** Summary of compound reactivities with lipid membranes composed of DOPC or DOPC/DOPS (4:1). All experiments were conducted at 37°C, pH 7.4, [lipid] = 1.27 mM, [compound] = 127 μM, and were analyzed after 24 hours unless otherwise indicated. Lysolipid concentration data are provided in table S2. OPC concentration changes are determined with reference to a control without compound, allowing for an experimental error of 20% (determined from replicate measurements). “Clear” lipidation signifies that lipidated compounds were clearly visible in the LC-MS analysis after 24 hours, with corroborating data available. Corroborating data include MS/MS fragmentation, comparison with authentic samples, or expected relative retention times for oleoyl and palmitoyl species in experiments with 1-palmitoyl-2-oleoyl-*sn*-glycero-3-phosphocholine (POPC). Compounds underlined gave notably strong ion intensities (>10^4^) for the lipidated derivative. “Potential” lipidation signifies that a peak with correct mass/charge ratio (*m*/*z*) was detected in LC-MS analysis, sometimes after 3 days, but without corroborating data.

**Lipid**	**[OPC] change**	**Lipidation**		
		**Clear**	**Potential**	**No evidence**
**DOPC**	Increase	**10h**, **11b**, 21*	**8**, **10j**, **12**	**11c**†, 15*, 22*†
	None	**10i**, **17**	**3**, **10d**, 18*, **20**	**1**, **2**, **4–7**, **10b**, **10c**, **10e-g**, **11a**, **11d**, **13**, **16**, **19**
	Decrease	**9a**, **9b**, **9c**, **9d**, **9e**, **10a**, **14**	–	–
DOPC + DOPS (4:1)	Increase	**9e**, **10a**, **10i**, **11b**	**3**, **8**, **10j**	**11a**, **11c**†, **11d**, **13**, **16**
	None	**9a**, **9b**, **9c**, **9d**, **10h**, **14**, **17**	**10d**, **12**, 18*, **19**	**1**, **2**, **4–7**, **10b**, **10c**, **10e-g**, 15*
	Decrease	21*	**20**	22*

*Samples analyzed after 72 hours.

†Notably high [OPC].

### Initial screens for reactivity

From Table 1, it is immediately clear that the full range of compound reactivity with lipids is exhibited, with some molecules, such as **9a-e**, providing clear evidence of considerable reactivity and others, such as **10e-g**, giving no discernable change. Of the 38 compounds tested, 12 were found to undergo unambiguous lipidation, with 9 of these producing high ion intensities for the lipidated compound. Of these nine, five belong to one class of compound (indazole, **9a-e**) and three to a related class of compound (benzimidazole, **10a**, **10h**, and **10i**). It is clear therefore that lipidation activity has a structural dependence.

**Fig. 3:**
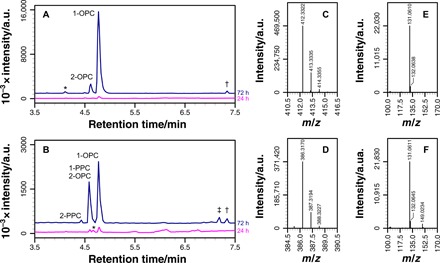
Reaction profiles for compound 9e with DOPC and POPC. In all cases, † identifies the peak corresponding to oleoyl-**9e**, ‡ identifies the peak corresponding to palmitoyl-**9e**, and impurities are identified by asterisks. 1-PPC and 2-PPC correspond respectively to 1-palmitoyl-*sn*-glycero-3-phosphocholine and 2-palmitoyl-*sn*-glycero-3-phosphocholine. (**A** and **B**) Base peak chromatograms (*m*/*z* range, 100 to 650) after **9e** addition to DOPC (A) or POPC (B) membranes after 24 and 72 hours. Chromatograms after 72 hours have been offset on the *y* axis by an arbitrary amount. a.u., arbitrary units. (**C**) Mass spectrum of oleoyl-**9e** from (A) (theoretical *m*/*z* [M + H]^+^, 412.3312). (**D**) Mass spectrum of palmitoyl-**9e** from (B) (theoretical *m*/*z* [M + H]^+^, 386.3156). (**E** and **F**) Tandem mass spectra resulting from the fragmentation of the [M + H]^+^ ions for oleoyl-**9e** and palmitoyl-**9e** from (C) and (D), respectively.

**Fig. 4:**
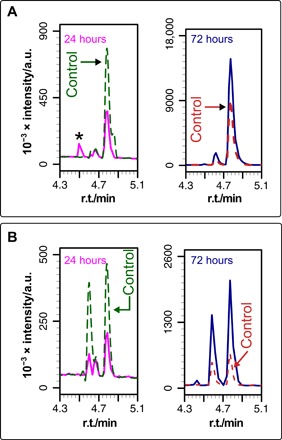
Lysolipid formation during incubation of compound 9e with DOPC and POPC. Expanded views of the base peak chromatograms (*m*/*z* range, 100 to 650) from LC-MS analysis of mixtures of **9e** with DOPC (**A**) or POPC (**B**) liposomes. Data from the corresponding blanks (DOPC or POPC without the addition of **9e**) are shown as dashed lines. r.t., retention time. The peak marked with an asterisk is an impurity.

Compound **9e** (Figs. 3 and 4) demonstrates the complex relationship between lipidation and lysolipid formation exhibited by many of the compounds for which there is clear lipidation. In the initial periods following the addition of **9e** to neutral liposomes [DOPC and 1-palmitoyl-2-oleoyl-sn-glycero-3-phosphocholine (POPC)], the concentration of lysolipid reduces in comparison to a control sample without **9e**. Two potential reasons for this decrease are that either **9e** binding modifies membrane properties in such a manner that the rate of lipid hydrolysis is reduced, or **9e** is more reactive toward lysolipids than lipids. After longer time periods in the presence of **9e**, the extent of lysolipid formation eventually surpasses lysolipid generation in the control sample.

### Relationship between lysolipid formation and lipidation

Across the range of compounds that undergo demonstrable lipidation in DOPC, all possible types of simultaneous lysolipid change relative to control samples are seen, from significant increases for **10h** and **21** to an almost complete absence of lysolipid for compound **9b**. Decreases in lysolipid concentration in DOPC membranes are restricted to compounds that undergo lipidation. For compounds that yield no discernable lipidation products, there is either no change in lysolipid levels or an increase. Overall, the data are sufficient to indicate that there is no clear relationship between the change in lysolipid concentration and lipidation activity.

In DOPC and DOPC/DOPS membranes, a number of compounds yielded significant increases in the concentration of oleoyl phosphatidylcholine (OPC) without concomitant detection of a lipidated product. In these cases, it must be assumed that lipid hydrolysis arises via the catalytic route in Fig. 1. Compounds **11a** to **11d** were generally effective at increasing lysolipid formation in most systems, yet, of these, only **11b** generates a detectable lipidation product. A comparison of compounds **11c**, **10i**, and **17** is revealing because, of these, **11c** generates lysolipid without lipidation, whereas the other two generate lipidation without generating excess lysolipid. All three of these compounds have the same aminopropyl group, which suggests that some of their reactivity is dictated by the partitioning behavior of the aromatic group in the membrane interface.

It should be noted that the hydrolysis and acyl transfer (lipidation) reactions differ in their ability to generate lysolipid. The hydrolysis reaction is catalytic and therefore has the potential to generate a large excess of lysolipid with respect to the quantity of compound present. Conversely, if a compound exhibits lipidation activity alone (i.e., without concomitant changes in the levels of hydrolysis), then the maximum quantity of lysolipid formed will be equivalent to the quantity of compound that has participated in the acyl transfer reaction. A consequence of this 1:1 stoichiometry is that, in many cases, even where an acyl transfer reaction has gone to completion and the compound has been completely consumed, the quantity of lysolipid formed with respect to the total lipid will remain very small and may fall below the detection limit for many analytical methods.

### Correlation between reactivity and predicted molecular properties

Most compounds for which unambiguous lipidation is reported are amines for which the predicted ammonium ion p*K*_a_ is more than 1 pH unit greater than pH 7.4 (table S1), the pH at which the experiments were conducted. The exceptions are **9a** (predicted to be 8.16), **10h** (6.93), and **14** (3.79). These pH differentials, even allowing for differences in the p*K*_a_ between the membrane-associated form and the form in solution, imply that, for most compounds, the lipidation reaction does not require the amino form of the compound to be predominant at the pH of the experiment. The compounds that undergo unambiguousx lipidation have predicted log*P* values in the range of −1.19 (**9e**) to 2.26 (**21**), with the majority being close to 1 (table S1). Their log*D* values cover the range of −1.63 to 0.72, with most being <0. Therefore, with the possible exception of **21**, none would normally be considered to be sufficiently hydrophobic that they would be expected to partition into lipid membranes with high affinity.

### Reaction selectivity

In DOPC/DOPS membranes, lipidation is found with the same compounds that are reactive with DOPC but with subtle changes in reactivity. Notably, the reduction in lysolipid levels after 24 hours for many of the most active compounds in DOPC is generally not seen in DOPC/DOPS. This observation is significant because it demonstrates selectivity at the level of membrane composition. In addition, in all samples where lyso-phosphatidylcholine (lyso-PC)was formed, lyso-phosphatidylserine (lyso-PS) was also generated, which would be significant if replicated in vivo given the high biological activity of lysolipids (*17*) and the particular role of lyso-PS in the immune response (*19*).

Compounds **10a** and **10d to 10g** form a homologous series from a primary amino compound (**10a**) through a series of secondary amines of increasing steric bulk. Of this series, **10a** produces significant evidence of lipidation, **10d** is potentially lipidated, but the evidence for lipidated product formation is tentative, and none of the others yield any evidence of lipidation or increased formation of oleoyl PC. When comparing similarly homologous pairings of a primary amine with its *N*-ethyl secondary amine equivalent, such as compounds **10h** (strong lipidation) versus **10j** (potentially weak lipidation) and **11b** (lipidation) versus **11d** (no lipidation), it becomes apparent that steric bulk close to the nucleophilic center significantly reduces the aminolysis activity.

To probe the selectivity for acyl chain transfer, a selection of compounds, composed mostly of examples for which there was clear lipidation in DOPC, was examined further in membranes composed of POPC or 1-oleoyl-2-palmitoyl-*sn*-glycero-3-phosphocholine (OPPC). The lipidation data for these are in Table 2 (lysolipid formation) and tables S3 to S7 (spectra of lipidated derivatives). In general, the data for POPC and OPPC are as anticipated on the basis of the prior experiments with DOPC, with typically <5–parts per million error between the observed and predicted mass/charge ratio (*m*/*z*) for [M + H]^+^ for each palmitoylated and oleoylated compound. Many of the lipidated amines yielded fragmentation patterns in accordance with those expected for amides, with cleavage of the N─C(alkyl) bond particularly prevalent for species able to form benzylic cations (*20*). For example, both oleoyl (Fig. 3C) and palmitoyl (Fig. 3D) derivatives of **9e** yielded the same fragment with *m*/*z* 131.1 ([indazole─CH_2_]^+^; Fig. 3, E and F) when the parent [M + H]^+^ ions (*m*/*z* 412.3 and 386.3, respectively) were fragmented by collision-induced decay.

**Table 2:** Lysolipid formation in membranes composed of POPC or OPPC in the presence of low–molecular weight organic compounds. Errors are ± σ (*n* = 3). All values given those after subtraction of the OPC concentration in a control without compound incubated in the same conditions. For POPC, the concentrations in controls (without compound) after 24 hours were as follows: [OPC], 0.013 ± 0.004 mM; [PPC], 0.010 ± 0.003 mM. For OPPC, the concentrations in controls after 24 hours were as follows: [OPC], 0.006 ± 0.001 mM; [PPC], 0.006 ± 0.002 mM.

**Compound**	**POPC**		**OPPC**	
	**[OPC] change after** **24 hours/mM**	**[PPC] change after** **24 hours/mM**	**[OPC] change after** **24 hours/mM**	**[PPC] change after** **24 hours/mM**
**3**	−0.002 ± 0.005	−0.003 ± 0.004	−0.002 ± 0.002	−0.001 ± 0.003
**8**	0.013 ± 0.007	0.013 ± 0.007	0.011 ± 0.004	0.012 ± 0.005
**9a**	−0.012 ± 0.004	−0.010 ± 0.004	−0.003 ± 0.001	−0.002 ± 0.002
**9b**	−0.012 ± 0.004	−0.010 ± 0.004	0.000 ± 0.002	0.000 ± 0.002
**9c**	−0.013 ± 0.004	−0.010 ± 0.004	−0.001 ± 0.002	−0.001 ± 0.002
**9d**	−0.006 ± 0.005	−0.006 ± 0.005	−0.001 ± 0.002	−0.001 ± 0.002
**9e**	−0.009 ± 0.005	−0.009 ± 0.004	−0.005 ± 0.001	−0.004 ± 0.002
**10a**	−0.006 ± 0.005	−0.007 ± 0.004	−0.004 ± 0.001	−0.002 ± 0.002
**10h**	0.047 ± 0.014	0.034 ± 0.010	0.007 ± 0.003	0.007 ± 0.004
**10i**	0.017 ± 0.008	0.011 ± 0.006	0.004 ± 0.003	0.028 ± 0.008
**10j**	0.025 ± 0.010	0.015 ± 0.007	0.010 ± 0.004	0.008 ± 0.004
**11a**	0.039 ± 0.012	0.026 ± 0.009	0.001 ± 0.002	0.002 ± 0.003
**11b**	0.027 ± 0.010	0.015 ± 0.007	0.065 ± 0.014	0.060 ± 0.014
**11c**	0.033 ± 0.011	0.016 ± 0.007	0.001 ± 0.002	0.000 ± 0.003
**11d**	0.027 ± 0.010	0.016 ± 0.007	0.001 ± 0.002	0.001 ± 0.003
**13**	−0.003 ± 0.005	−0.002 ± 0.004	0.000 ± 0.002	0.001 ± 0.003
**14**	0.007 ± 0.006	0.005 ± 0.005	0.021 ± 0.006	0.023 ± 0.007
**16**	0.014 ± 0.008	0.011 ± 0.006	0.004 ± 0.003	0.002 ± 0.003
**17**	0.008 ± 0.007	0.007 ± 0.006	0.014 ± 0.005	0.014 ± 0.005
**19**	−0.006 ± 0.004	−0.004 ± 0.004	−0.002 ± 0.002	−0.002 ± 0.003
**20**	0.010 ± 0.007	0.007 ± 0.006	−0.004 ± 0.002	−0.004 ± 0.002

Across the sample set as a whole, no compounds exhibited absolute selectivity for the formation of a particular acylated product, although compound **8** (table S3) was unique in having a very high selectivity for formation of the oleoyl derivative in both OPPC and POPC membranes. Some of the compounds in the indazole series (notably **9b-d**) formed the oleoyl derivative ahead of the palmitoyl derivative.

## DISCUSSION

Agents that are capable of binding to membranes fall into one of two categories: Either they have no effect on lipid stability, or they are able to moderate lysis by acting as fatty acyl acceptors or catalyzing hydrolysis. It has previously been demonstrated that lipidation of the β-blocker propranolol yields a product that disrupts the membrane integrity of liposomes and has surfactant properties, with a critical micelle concentration of approximately 10^−5^ M (*13*). Compounds that induce lysolipid formation have also been linked with idiosyncratic toxic effects in vivo such as drug-induced phospholipidosis (*13*, *14*). It is likely, therefore, that many agents that affect lipid stability have similar activity. High membrane affinity is not a prerequisite for high lytic activity. Agents that catalyze hydrolysis require at least one center that is acidic or basic and is presumably located in the interfacial region in the membrane-associated form. Acyl group acceptors require the presence of at least one nucleophilic center that is suitably disposed for reaction with a lipid carbonyl group. Of the agents that are able to serve as acyl group acceptors, many show a modest selectivity for the acyl derivative formed. This acyl selectivity, alongside the change in lysolipid concentration, can be used to classify the compounds.

### Classification of compounds that undergo lipidation

Compounds can be classified in one of three general ways according to their behavior in DOPC, POPC, and OPPC.

Type I compounds, typified by **8** (table S3), **10h-j** (tables S6 to S7), **11a-d**, **14**, **16**, **17**, and **20**, produce an increase in the concentrations of OPC and palmitoyl phosphatidylcholine (PPC) after 24 hours in comparison to controls, either through a higher aminolysis reactivity with lipids than lysolipids or because they increase the rate of lipid hydrolysis. For most cases in POPC membranes, the increase in OPC concentration is greater than that of PPC, whereas for OPPC, the increases in OPC and PPC concentration are similar. Examination of the extracted ion chromatograms for the lipidated species reveals that most of these compounds display some selectivity, albeit very small, for the acyl group transferred to the compound. The underlying mechanisms for this selectivity are likely to be complex. Compound **10h**, for example, preferentially forms the oleoyl-modified species in POPC membranes and the palmitoyl-modified species in OPPC membranes (table S6), while producing the changes in lysolipid described above that favor OPC formation in POPC and equal formation of OPC and PPC in OPPC (Table 2). In contrast, compound **10i** is notable in favoring the formation of the palmitoyl-modified product in both POPC and OPPC membranes (table S7), while producing low levels of OPC formation alongside significant PPC formation in the case of OPPC membranes. These patterns of product formation can only be rationalized by considering aminolysis reactivity with the lipid and the lysolipids alongside hydrolysis activity catalyzed by either the unmodified or the lipidated compound (or both). Consistent with this complex lytic mechanism, an increased abundance of the ion corresponding to the 3-phosphoglycerol derivative glycerophosphocholine (GPC; Fig. 1 and figs. S2 and S3), formed by lysis of both acyl chains, was detectable in many experiments with lipidation type 1 compounds. As the chromatography was optimized for less polar compounds and GPC was therefore in the injection peak, only a semiquantitative assessment, however, is merited.

Type II compounds, including **9a-d** (table S4), **9e** (Fig. 3), **10a** (table S5), and **19**, exhibit a reduction in the concentrations of OPC and PPC in comparison to control samples after 24 hours. In principle, this reduction can arise either because the compound has a higher aminolysis reactivity with the lysolipid than the lipid or because the compound reduces rate of background hydrolysis. The latter mechanism would appear most likely as the levels of GPC remain the same or even decrease after 24 hours in the presence of these compounds (fig. S2). There is no observed difference in the reduction in OPC versus PPC, but there is evidence of some selectivity in the chromatographic profiles for the lipidated species (tables S4 and S5). Compound **9a**, for example, exhibits an increased formation of oleoylated compound in comparison to the palmitoylated species in POPC, whereas in OPPC, the palmitoylated species is in greater abundance. Given the low formation of GPC, it is likely that this small degree of acyl selectivity arises from a preferential reaction at the *sn*-2 acyl group of the lipid.

Type III compounds, typified by **3** and **13**, produce little change in the levels of OPC and PPC in comparison to controls, either because the level of background reactivity is small or the rate of lysolipid generation by hydrolysis and aminolysis is matched by the rate of lysolipid consumption by aminolysis.

## CONCLUSIONS

Our data demonstrate that (i) low–molecular weight molecules with low predicted log*P*/log*D* partake in membrane-lytic reactions. A strong membrane interaction is not a requirement for lytic activity; (ii) the reactivity exhibits selectivity according to both the chemical structure of the molecule interacting with the membrane and the chemical composition of the membrane. Therefore, in principle, it should be possible to predict or design such activity, although the rules for these processes are still not fully understood; and (iii) the formation of lysolipids is not a reliable measure of the lytic potential of a given compound.

A more thorough understanding of the patterns of reactivity exhibited by membrane-associating molecules decorated with nucleophilic centers, such as the aromatic amines, alcohols, and thiols used in this study, will require detailed mechanistic studies to delineate the relative importance of membrane interfacial binding depth and orientation, interfacial water activity, and structural parameters such as the presence of internal basic sites.

## MATERIALS AND METHODS

### Materials

DOPC, POPC, OPPC, DOPS, 1-OPC, and 1-palmitoyl-*sn*-glycero-3-phosphocholine (1-PPC) were purchased as powders from Avanti Polar Lipids (via INstruchemie B.V., The Netherlands or Sigma-Aldrich, Dorset, UK). Compounds **2** (A00.537.799), **3** (K00.128.576), **4** (A00.388.211), 5 (K02.783.027), **6** (A00.788.063), **9a** (A05.891.682), **9b** (A06.826.360), **9c** (A06.788.730), **9d** (A02.626.646), **9e** (A06.826.359), **10h** (K00.644.797), **10a** (K02.035.210), **10i** (K00.242.288), **10j** (A01.480.727), **11a** (A00.739.562), **11b** (A00.000.070), **11c** (K01.793.053), **11d** (A05.884.302), **12** (A00.704.412), **13** (A00.152.963), **17** (A00.064.429), **19** (A01.075.774), and **20** (A01.072.662) were purchased from Aurora Fine Chemicals (San Diego, CA 92126, USA) (product numbers at time of purchase in parentheses).

Compounds **14** (FCH1116113), **15** (FCH867785), and **16** (FCH998721) were purchased from FCH, Chernigiv, Ukraine (product numbers at time of purchase in parentheses).

Other compounds and solvents were purchased from Sigma-Aldrich (Dorset, UK), Fluorochem (Hadfield, UK), Alfa Aesar (Heysham, UK), or Fisher Scientific (Loughborough, UK).

### Liposome preparation

Liposomes were prepared by drying a solution of the lipid from a solution in chloroform to form a thin film around the side of a round-bottomed flask. This film was then hydrated with buffer and, after thorough vortex mixing, was subjected to five freeze-thaw cycles using liquid nitrogen and a warm water bath (40°C). The vesicles were then extruded 10× through laser-etched polycarbonate membranes (Whatman; 100-nm pore size) at 50°C using a thermobarrel extruder (Northern Lipids, Burnaby, Canada) under a positive pressure of N_2_.

### Lipidation in liposomes

All liposome experiments were conducted at a lipid concentration of 1.27 mM. Compounds tested for membrane-lytic activity were used at a concentration of 0.127 mM. Samples at pH 7.4 were buffered using 10 mM sodium bicarbonate and contained NaCl at a concentration of 90 mM. Samples were made by adding compound solutions to preformed liposome dispersions before incubation in a sealed vial in either a temperature controlled thermal block or a thermostated shaking incubator. For analysis by LC-MS, a small volume (typically <20 μl) of the reaction mixture was removed and diluted into MeCN/H_2_O (1:1) in a sample vial to give a drug concentration of 1 μg/ml. The sample injection volume was 3 μl.

### Liquid chromatography–mass spectrometry

LC-MS and LC–tandem MS (MS/MS) data were acquired on a SYNAPT G2-S (Waters Corporation, UK) instrument, with time-of-flight analyzer recording electrospray ionization ions in the range of 50 to 2000 *m*/*z* and a scan time of 1.0 s. For the analysis of mixtures of compounds with liposomes, chromatography was conducted using a 3-μl sample injection on to an Acquity Ultra Performance Liquid Chromatography (UPLC) equipped with a BEH Phenyl 1.7 μm; 2.1 mm by 50 mm) column. The flow rate was 0.4 ml/min. The solvent gradient (A:B), using H_2_O (A) and MeCN (B), with both solvents containing 0.1% formic acid, was either (gradient 1): 95:5 over 0.5 min (isocratic), 95:5 to 30:70 over 2.5 min, 30:70 to 5:95 over 4 min, 5:95 (isocratic) for 1 min, 5:95 to 95:5 over 0.5 min, 95:5 (isocratic) for 0.5 min; or (gradient 2): 95:5 over 0.5 min (isocratic), 95:5 to 5:95 over 7 min, 5:95 (isocratic) for 1.3 min, 5:95 to 95:5 over 0.1 min, 95:5 (isocratic) for 1.1 min.

All solvent transitions used linear gradients. The electrospray parameters were as follows: capillary voltage, 1 kV; source temperature, 150°C; sampling cone voltage, 50 V; source offset voltage, 30 V; desolvation temperature, 350°C; cone gas flow, 60 liters hour^−1^; desolvation gas flow, 600 liters hour^−1^; and nebulizer gas flow, 6 bar.

Collision-induced dissociation (CID) MS/MS was carried out in the trap region of a SYNAPT G2-S (Waters Ltd., UK). Desired precursor ions were isolated in the quadrapole and subsequently underwent CID fragmentation ramping from 30 to 50 V.

The data were processed using MassLynx software (version 4.1 SCN924), MZmine (version 2.38), (*21*) and the xcms LC-MS and gas chromatography–MS data analysis package (version 1.52.0) (*22*) in the R statistical computing environment (version 3.4.1) (*23*).

### Calibration curves

Signal responses were determined by the least-squares fitting of Exponentially Modified Gaussian functions (*24*) to peak profiles from the extracted ion chromatograms (*m*/*z* for the monoisotopic molecular ion, *z* = 1, ±0.005) of the ions of interest. A standard curve for signal response in relation to lysolipid concentration was generated by least-squares fitting of a general logistic model (Eq. 1) to data obtained using an authentic standard of OPC at known injection concentrations.

$$A_{\text{calc}}=A_{u}/1+e^{-s(c-c_{0.5})}$$ (1)

where *A*_calc_ is the calculated value, *A*_u_ is the maximum area, *s* is the steepness of the curve, *c* is the natural logarithm of the analyte concentration, and *c*_0.5_ is the natural logarithm of the concentration at half maximum. Fitting data are presented in fig. S1.

### Molecular information

Molecular properties were calculated using Advanced Chemistry Development (ACD) Labs software accessed through Chemical Abstracts Service/SciFinder (*18*). Values for p*K*_a_ were calculated at 25°C and zero ionic strength in aqueous solutions for the most acidic or most basic sites in the molecule (with the p*K*_a_ for the most basic site in the protonated form). Values for log*P* and log*D* were calculated at pH 7 and 25°C (in the neutral form for log*P* and the predominant predicted ionization state at this pH for log*D*).

## Supplementary Material

aaz8598_SM.pdf

## SUPPLEMENTARY MATERIALS

Supplementary material for this article is available at http://advances.sciencemag.org/cgi/content/full/6/17/eaaz8598/DC1

## Appendix

View/request a protocol for this paper from *Bio-protocol*.
