# Supplementary material for: Lysis of membrane lipids promoted by small organic molecules: Reactivity depends on structure but not lipophilicity
Source: Sci Adv. 2020 Apr 22;6(17):eaaz8598. doi: 10.1126/sciadv.aaz8598 (PMC7176411; doi:10.1126/sciadv.aaz8598)
Supplement: aaz8598_SM.pdf [file aaz8598_SM.pdf]

Science Advances
AAAS

advances.sciencemag.org/cgi/content/full/6/17/eaaz8598/DC1

Supplementary Materials for

Lysis of membrane lipids promoted by small organic molecules: Reactivity depends on structure but not lipophilicity

Hannah M. Britt, Aruna S. Prakash, Sanna Appleby, Jackie A. Mosely, John M. Sanderson*

*Corresponding author. Email: j.m.sanderson@durham.ac.uk

Published 22 April 2020, *Sci. Adv.* **6**, eaaz8598 (2020)
DOI: 10.1126/sciadv.aaz8598

This PDF file includes:

Synthetic procedures and compound characterization
Figs. S1 to S3
Tables S1 to S7
References

SUPPLEMENTARY MATERIALS

Synthetic Procedures

General Methods. Dry solvents were sourced from Fisher Scientific (Loughborough, UK) and Sigma-Aldrich (Dorset, UK), with the exception of dichloromethane (DCM), which was distilled from calcium hydride immediately before use. Other reagents, including 2-(chloromethyl)benzimidazole, thioglycolic acid, 2-Aminomethylbenzimidazole and *o*-phenylenediamine, were obtained from Sigma-Aldrich, UK. Purification was performed by flash column chromatography using a silica gel support (230–400 mesh, 40–60 μm) from Sigma–Aldrich (UK). Thin layer chromatography (TLC) was conduced using aluminium backed silica gel 60 F_{254} TLC plates (Merck Millipore, UK). TLC plates were visualized using either an ultraviolet (UV) lamp (254 nm), iodine chamber or by use of a dipping stain followed by heating. Dipping stains used included, phosphomolybdic acid (PMA) and $KMnO_4$. PMA stain consisted of PMA (6 g) and $Ce(SO_4)_2.4H_2O$ (3 g) dissolved in H_2SO_4 (15 ml) and H_2O (230 ml). $KMnO_4$ stain was prepared by dissolving $KMnO_4$ (1.5 g) and K_2CO_3 (10 g) in 10% NaOH solution (1 ml) and H_2O (200 ml). Distillation was performed using a Buchi Glass Oven B-585 Kugelrohr operating at a pressure between 0.2- 2.0 Torr. NMR data were collected on a Bruker Avance-400 (at 400 MHz for ^{1}H; 100.6 MHz for ^{13}C) or Varian VNMRS (at 700 MHz for ^{1}H; 176 MHz for ^{13}C). NMR spectra were obtained in $CDCl_3$ (97% D, Goss Scientific Instruments ltd., Cheshire, UK) and are reported in ppm using residual $CHCl_3$ at 7.26 ppm as the internal reference. ^{13}C NMR spectra are referenced to solvent as internal reference (77.23 ppm for $CDCl_3$). NMR data were processed using Mestrenova (Mestralab Research, version 10.0). Routine accurate mass characterisation was conducted on a LCT Premier XE (Waters Corp., UK) equipped with an Atmospheric Solids Analysis Probe (ASAP) ion source or QToF Premier (Waters Corp., UK). IR spectroscopy was performed on a Perkin Elmer Paragon 1000 FT-IR spectrometer with an ATR attachment operating through the wavenumber range 400 cm^{-1} and 4000 cm^{-1}. IR data were processed using the instrument software.

Synthesis of 2-(mercaptomethyl)benzimidazole (10c).(*25*, *26*)

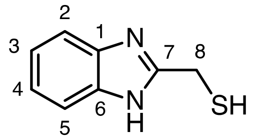

A mixture of thioglycolic acid (1.40 ml, 20.0 mmol) and *o*-phenylenediamine (1.89 g, 17.5 mmol) was refluxed in 37% HCl(aq) (15 ml) for 12 hours. Aqueous solvent was removed by freeze drying, and MeOH recrystallization of the residue yielded the HCl salt of **10c** as a white powder (2.61 g, 91%): ^{1}H (400 MHz; DMSO-d6) δ 4.18 (s, 2H, H_8), 7.19 (dd, J = 3.2, 5.9 Hz, 2H, $H_{3,4}$), 7.53

(dd, J = 3.2, 5.9 Hz, 2H, $H_{2,5}$); ^{13}C (100 MHz; DMSO-d6) δ 36.1 (C_8), 115.4 ($C_{2,5}$), 122.3 ($C_{3,4}$), 139.7 ($C_{1,6}$), 151.0 (C_7); IR (neat) v_{max}/cm^{-1} 1624, 2879, 2946, 2995, 3155; LRMS (ESI) *m*/*z* 165.0 [M+H]$^+$; HRMS (ESI) calculated for $C_8H_9N_2S$ [M+H]$^+$ 165.0486, found 165.0474.

Synthesis of 2-((methylamino)methyl)benzimidazole (10d).(*27*, *28*)

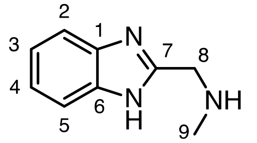

Finely powdered 2-(chloromethyl)benzimidazole (0.46 g, 1.64 mmol) was added to 33% methylamine in EtOH (9 ml) at 0 °C. The solution was stirred at 0 °C for 45 minutes before the solution was allowed to warm to room temperature over 15 minutes. After dilution with H_2O (20 ml), the mixture was extracted with CH_2Cl_2 (3 × 20 ml). The combined organic extracts were dried over $MgSO_4$, and after removal of the drying agent by filtration, the solvent was removed *in vacuo*. Kugelrohr distillation under reduced pressure yielded compound **10d** as a yellow oil (0.18g, 69%): ^{1}H (400 MHz; DMSO-d6) δ 2.38 (s, 3H, H_9), 4.03 (s, 2H, H_8), 7.19-7.22 (m, 2H, $H_{3,4}$), 7.50-7.56 (m, 2H, $H_{2,5}$), 12.13 (s, 1H, NH); ^{13}C (100 MHz; DMSO-d6) δ 34.8 (C_9), 48.1 (C_8), 114.8 ($C_{2,5}$), 123.0 ($C_{3,4}$), 138.1 ($C_{1,6}$), 151.3 (C_7); IR (neat) v_{max}/cm^{-1} 1626, 2855, 2999, 3065; LRMS (ASAP) *m/z* 162.1 $[M+H]^+$; HRMS (ASAP) calculated for $C_9H_{12}N_3$ $[M+H]^+$ 162.1031, found 162.1027.

Synthesis of 2-((ethylamino)methyl)benzimidazole (10e).(*27*, *28*)

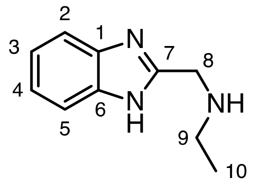

The protocol used for the preparation of **10d** was used, with ethylamine (3 ml in EtOH (6 ml) in place of methylamine, to yield the title compound as a yellow oil (0.20 g, 71%): ^{1}H (400 MHz; DMSO-d6) δ 1.32 (t, J = 8.0 Hz, 3H, H_{10}), 2.68 (q, J = 8.0 Hz, 2H, H_9), 4.04 (s, 2H, H_8), 7.21-7.24 (m, 2H, $H_{3,4}$), 7.50-7.55 (m, 2H, $H_{2,5}$), 12.13 (s, 1H, NH); ^{13}C (100 MHz; DMSO-d6) δ 14.0 (C_{10}), 42.3 (C_9), 47.9 (C_8), 115.3 ($C_{2,5}$), 123.0 ($C_{3,4}$), 138.2 ($C_{1,6}$), 149.3 (C_7); IR (neat) v_{max}/cm^{-1} 1626, 2858, 3000, 3065; LRMS (ESI) *m/z* 176.1 $[M+H]^+$; HRMS (ESI) calculated for $C_{10}H_{14}N_3$ $[M+H]^+$ 176.1188, found 176.1186.

Synthesis of 2-((propylamino)methyl)benzimidazole (10f).(*27*, *28*)

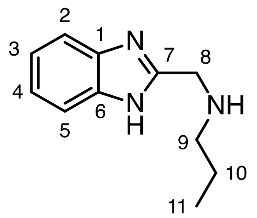

The protocol used for the preparation of **10d** was used, with propylamine (3 ml in EtOH (6 ml) in place of methylamine, to yield the title compound as a yellow oil (0.21 g, 68%): ^{1}H (400 MHz; DMSO-d6) δ 1.29 (t, J = 8.0 Hz, 3H, H_{11}), 1.49-1.58 (m, 2H, H_{10}), 2.75 (t, J = 8.0 Hz, 2H, H_9), 4.01 (s, 2H, H_8), 7.15-7.19 (m, 2H, $H_{3,4}$), 7.52-7.58 (m, 2H, $H_{2,5}$), 12.29 (s, 1H, NH); ^{13}C (100 MHz; DMSO-d6) δ 14.1 (C_{11}), 29.8 (C_{10}), 42.3 (C_9), 47.8 (C_8), 115.3 ($C_{2,5}$), 122.0 ($C_{3,4}$), 138.2 ($C_{1,6}$), 149.3 (C_7); IR (neat) v_{max}/cm^{-1} 1630, 2853, 3017, 3078; LRMS (ASAP) *m/z* 190.1 $[M+H]^+$; HRMS (ASAP) calculated for $C_{11}H_{16}N_3$ $[M+H]^+$ 190.1344, found 190.1343.

Synthesis of 2-((isopropylamino)methyl)benzimidazole (10g).(*27*, *28*)

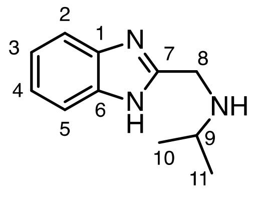

The protocol used for the preparation of **10d** was used, with isopropylamine (3 ml in EtOH (6 ml) in place of methylamine, to yield the title compound as a yellow oil (0.18 g, 59%): ^{1}H (400 MHz; δ 1.27 (d, *J* = 8.0 Hz, 6H, $H_{10,11}$), 2.78 (sept, *J* = 8.0 Hz, 1H, H_9), 4.01 (s, 2H, H_8), 7.15-7.20 (m, 2H, $H_{3,}$4), 7.52-7.58 (m, 2H, $H_{2,5}$), 12.29 (s, 1H, NH); 1^{3}C (100 MHz; DMSO-d6) δ 15.0 ($C_{10,11}$), 42.3 (C_9), 47.8 (C_8), 115.3 ($C_{2,5}$), 122.0 ($C_{3,4}$), 138.2 ($C_{1,6}$), 149.3 (C_7); IR (neat) v_{max}/cm^{-1} 1631, 2855, 3008, 3077; LRMS (ASAP) *m/z* 190.1 [M+H]$^+$; HRMS (ASAP) calculated for $C_{11}H_{16}N_3$ [M+H]$^+$ 190.1344, found 190.1343.

Synthesis of N-((1*H*-benzimidazole-2-yl)methyl)oleamide (*N*-oleoyl 10a).

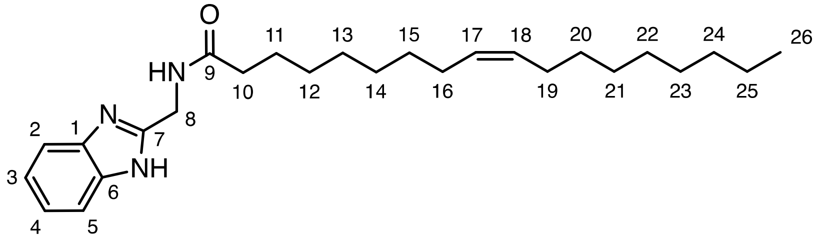

2-Aminomethylbenzimidazole (0.15 g, 1.02 mmol), pyridine (0.09 ml), 1.12 mmol), and oleoyl chloride (0.37 ml), 1.12 mmol) were stirred in dry CH_2Cl_2 (10 ml) under argon for 12 hours. The organic phase was washed with saturated NH_4Cl solution (3 × 10 ml), dried over $MgSO_4$, and after filtration the solvent removed *in vacuo*. Purification by Kugelrohr distillation yielded *N*-oleoyl **10a** as a yellow oil (0.26g, 61%): ^{1}H (400 MHz; DMSO-d6) δ 0.87 (t, 3H, *J* = 8.0 Hz, H_{26}), 1.23-1.28 (m, 20H, H_{Alkyl}), 1.63 (quin, 2H, *J* = 12.0 Hz, H_{11}), 1.95-2.01 (m, 4H, $H_{16,19}$), 2.27 (t, 2H, *J* = 8.0 Hz, H_{10}), 4.58 (m, 2H, H_8), 5.28-5.37 (m, 2H, $H_{17,18}$), 7.43-7.48 (m, 2H, $H_{3,4}$), 7.77-7.81 (m, 2H, $H_{2,5}$); ^{13}C (100 MHz; DMSO-d6) δ 14.1 (C_{26}), 22.6 (C_{Alkyl}), 25.6 (C_{Alkyl}), 27.1 (C_{Alkyl}), 27.2 (C_{Alkyl}), 29.1 (C_{Alkyl}), 29.2 (C_{Alkyl}), 29.3 (C_{Alkyl}), 29.5 (C_{Alkyl}), 29.7 (C_{Alkyl}), 31.9 (C_{Alkyl}), 36.3 (C_{10}), 37.9 (C_8), 122.7 ($C_{2,5}$), 129.6 ($C_{3,4}$), 129.8 ($C_{1,6}$), 130.0 ($C_{17,18}$), 152.0 (C_7) 175.8 (C_9); IR (neat) v_{max}/cm^{-1} 1635, 2930, 2995, 3347; LRMS (ESI) *m/z* 412.3 [M+H]$^+$; HRMS (ESI) calculated for $C_{26}H_{42}N_3O$ [M+H]$^+$ 412.3328, found 412.3325.

Synthesis of 4-[(1-oxo-octadec-9-enyl)amino]benzoic acid 2-(diethylamino)ethyl ester (*N*-oleoyl 21; *N*-oleoyl procaine).

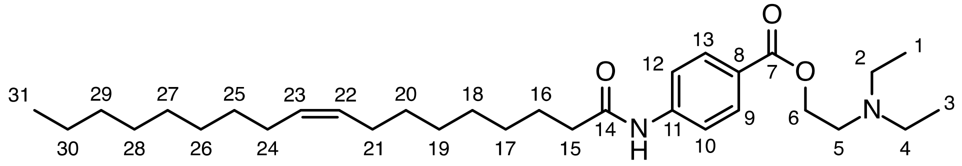

To procaine **21** (0.050g, 0.18 mmol) in dry CH_2Cl_2 (4 ml) was added pyridine (0.016 ml, 0.20 mmol) and oleoyl chloride 20 (0.061 ml, 0.20mmol). The mixture was stirred for 12 hours at room temperature, diluted with CH_2Cl_2 (6 ml) and washed with saturated NH_4Cl solution (3 × 10 ml). The organic layer was dried over $MgSO_4$ and filtered before the solvent was removed *in vacuo*. Purification by silica gel chromatography using EtOAc:MeOH:NH_3(14% aq.) (98:1:1) as eluent yielded *N*-oleoyl procaine as a yellow oil (0.063g, 70%): ^{1}H (400 MHz; $CDCl_3$) δ 0.87 (t, *J* = 8.0 Hz, 3H, H_{29}), 1.20-1.42 (m, 20H, H_{Alkyl}), 1.71 (quin, 2H, *J* = 12.0 Hz, H_{16}), 2.18-2.24 (m, 4H, $H_{21,24}$), 2.90 (t, 2H, $_J$ = 8.0 Hz, H_{15}), 3.00-3.10 (m, 4H, $H_{2,4}$), 3.20-3.25 (m, 2, H_5), 4.69-4.73 (m, 2H, H_6), 5.20-5.25 (m, 2H, $H_{22,23}$), 7.75 (d, *J* = 8.5 Hz, 2H, $H_{9,13}$), 8.0 (d, *J* = 8.5 Hz, 2H, $H_{10,12}$); ^{13}C (100 MHz; $CDCl_3$) δ 12.5 ($C_{1,3}$), 14.0 (C_{31}), 22.8 (C_{30}), 25.6 (C_{Alkyl}), 28.9 (C_{Alkyl}), 29.2 (C_{Alkyl}), 29.5 (C_{Alkyl}), 29.6 (C_{Alkyl}), 29.8 (C_{Alkyl}), 29.9 (C_{Alkyl}), 30.0 (C_{Alkyl}), 32.1 (C_{16}), 47.9 ($C_{2,4}$), 48.0 (C_{15}), 51.4 (C_5), 62.7 (C_6), 113.8 ($C_{10,12}$), 119.9 (C_8), 130.2 (C_{22}), 130.5 (C_{23}) 131.5 ($C_{9,13}$),

155.0 (C_{11}), 166.7 (C_7), 179.2 C_{14}); IR (neat) v_{max}/cm^{-1} 1598, 1602, 1720, 2855, 2925, 2985, 3010; LRMS (ESI) *m*/*z* 501.4 [M+H]$^+$; HRMS (ESI) calculated for $C_{31}H_{53}N_2O_3$ [M+H]$^+$ 501.4056, found 501.4051.

Supplementary Figures

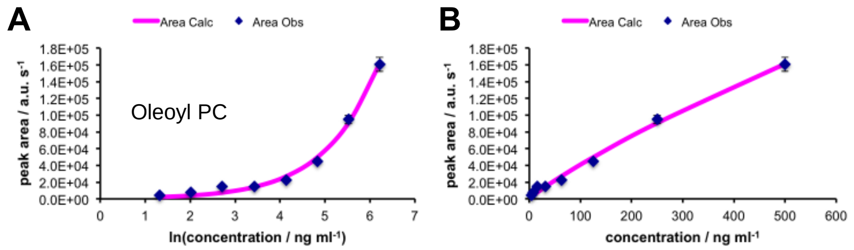

Fig. S1. Calibration curves showing the variation of chromatographic peak area response over a range of sample vial concentrations of authentic samples (with fixed injection volume). A/B, Oleoyl PC (summed areas for both 1- and 2-oleoyl). Raw data are shown as points, the outputs from fitting a general logistic model (Equation 1) to the data as lines. Each of the pair (**A** and **B**) shows the same data but differ in how the x-axis is plotted. Error bars are mean ± σ, n=3. Fitting parameters are: A_u, 1411744 a.u. s^{-1}; $c_{0.5}$, 8.46; s, 0.91; 8.4% error.

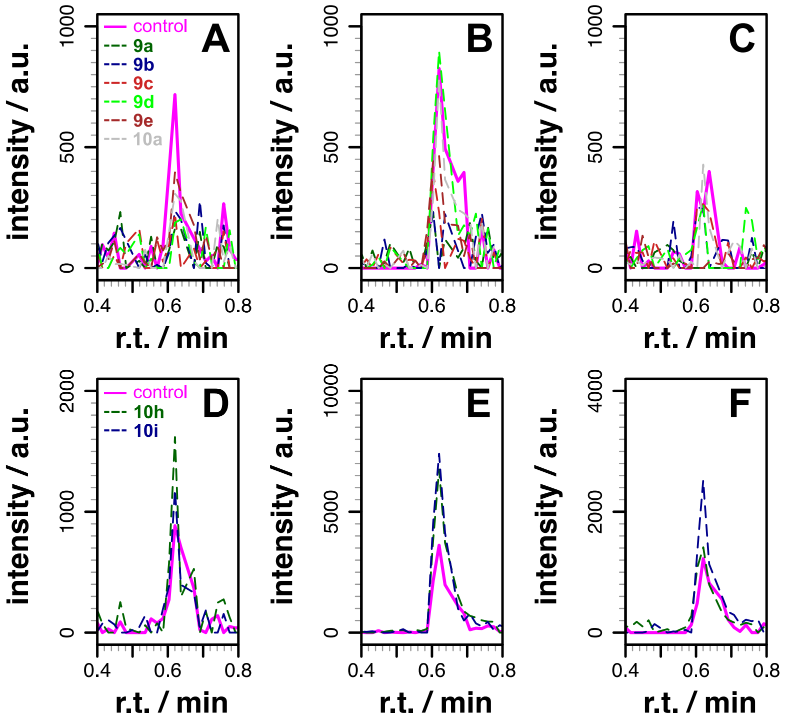

Fig. S2. Extracted ion chromatograms corresponding to *sn*-glycero-3-phosphocholine (GPC, m/z 258.110 ± 0.005). All chromatograms are from samples incubated for 24 h. The control samples (liposomes without compound) are shown as a solid magenta lines and samples that include compounds as dashed lines. **A-C**, **9a-e** and **10a** in liposomes composed of DOPC (**A**), POPC (**B**) and OPPC (**C**). **D-F**, **10h** and **10i** in liposomes composed of DOPC (**D**), POPC (**E**) and OPPC (**F**).

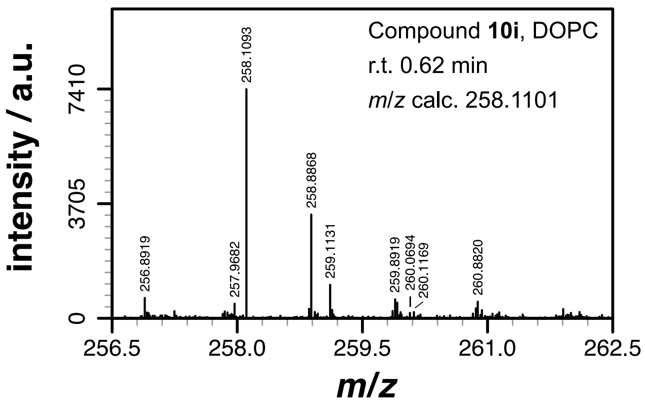

Fig. S3. Mass spectrum of s*n*-glycero-3-phosphocholine (GPC). The spectrum is from DOPC liposomes incubated with compound **10i**.

Supplementary Tables

Table S1. Properties of compounds used in this work. CAS numbers, molecular weights and log$P$ values are for the neutral compound. pK_a, log$P$, and log$D$ values are calculated at 25 °C and zero ionic strength in aqueous solutions for the most acidic or most basic sites in the molecule (with the pK_a for the most basic site in the protonated form) using Advanced Chemistry Development (ACD/Labs) Software V11.02 (© 1994-2019 ACD/Labs), accessed via the Chemical Abstracts Service/Scifinder (*18*). Log$D$ values are calculated at pH 7.

**Compound**	**CAS Number**	**M. Wt.**	**pK_a**	**log*P***	**log*D***
**1**	62-53-3	93.0579	4.61	1.14	1.13
**2**	5192-03-0	132.0687	4.94	0.85	0.85
**3**	874-05-5	133.0640	3.72	1.46	1.46
**4**	19335-11-6	133.0640	3.74	0.65	0.65
**5**	934-22-5	133.0640	6.10	0.28	0.23
**6**	18076-61-4	134.0592	3.00	0.96	0.96
**7**	134-32-7	143.0735	4.21	2.32	2.32
**8**	95-55-6	109.0528	4.74	0.50	0.49
**9a**	806640-37-9	147.0796	8.16	0.83	-0.61
**9b**	944898-72-0	147.0796	8.78	1.09	-0.94
**9c**	267413-25-2	147.0796	8.91	0.96	-1.12
**9d**	710943-26-3	147.0796	8.89	1.09	-0.98
**9e**	944904-20-5	147.0796	8.99	-1.19	-1.01
**10a**	5805-57-2	147.0796	12.24	0.87	-0.25
**10b**	4856-97-7	149.0589	11.57	0.95	0.95
**10c**	4344-85-8	164.0408	8.22	2.20	2.17
**10d**	98997-01-4	161.0953	12.45	1.43	0.22
**10e**	127855-46-3	175.1109	12.48	1.94	0.60
**10f**	342623-72-7	189.1266	12.48	2.45	1.20
**10g**	920464-24-0	189.1266	12.48	2.29	1.05
**10h**	934-32-7	133.0640	6.93	1.15	0.72
**10i**	42784-26-9	175.1109	11.91	1.68	-0.97
**10j**	21578-59-6	161.0953	7.26	2.04	1.37
**11a**	7250-19-3	132.0687	5.31	1.55	1.53
**11b**	22259-53-6	146.0844	10.48	1.61	-1.27
**11c**	6245-89-2	174.1157	10.63	1.81	-1.18
**11d**	57506-90-8	174.1157	10.86	2.28	-0.75
**12**	21109-25-1	146.0844	10.16	1.88	-1.04
**13**	95-23-8	149.0589	5.61	0.20	0.17
**14**	343868-71-3	160.0637	3.79	0.14	0.14
**15**	1027189-62-3	160.0637	2.58	-0.40	-0.40
**16**	431046-37-6	162.0793	3.96	1.22	1.22
**17**	79858-31-4	178.1106	9.85	0.89	-1.63
**18**	50-67-9	176.0950	9.52	0.54	-2.04
**19**	53718-45-9	240.0180	4.85	2.01	0.31
**20**	23375-15-7	240.0180	2.98	1.99	0.69
**21**	59-46-1	236.1525	9.24	2.26	0.05
**22**	94-24-6	264.1838	8.24	3.75	2.34

Table S2. Lysolipid formation in membranes composed of DOPC or DOPC/DOPS (4:1) in the presence of low molecular weight organic compounds. Errors are ± σ (n=3). All values given those after subtraction of the OPC concentration in a control without compound incubated in the same conditions. For DOPC, [OPC] in the control = 0.023 ± 0.006 mM after 24 h. For DOPC/DOPS, [OPC] in control = 0.019 ± 0.005 mM after 24 h.

Compound	[OPC] change after 24 h inubation with DOPC / mM	[OPC] change after 24 h incubation with DOPC/DOPS / mM
**1**	-0.001 ± 0.012	0.000 ± 0.022
**2**	-0.002 ± 0.015	-0.002 ± 0.014
**3**	0.009 ± 0.010	0.025 ± 0.010
**4**	0.002 ± 0.010	0.008 ± 0.011
**5**	0.003 ± 0.014	-0.002 ± 0.020
**6**	0.000 ± 0.012	-0.003 ± 0.014
**7**	-0.006 ± 0.009	-0.005 ± 0.009
**8**	0.035 ± 0.015	0.021 ± 0.010
**9a**	-0.018 ± 0.008	-0.001 ± 0.006
**9b**	-0.023 ± 0.007	-0.003 ± 0.006
**9c**	-0.015 ± 0.009	0.002 ± 0.007
**9d**	-0.018 ± 0.008	-0.002 ± 0.006
**9e**	-0.016 ± 0.009	0.018 ± 0.010
**10a**	-0.017 ± 0.008	0.016 ± 0.009
**10b**	0.000 ± 0.009	0.000 ± 0.005
**10c**	0.000 ± 0.008	0.002 ± 0.011
**10d**	0.002 ± 0.011	0.001 ± 0.007
**10e**	0.000 ± 0.009	-0.001 ± 0.006
**10f**	0.004 ± 0.007	0.004 ± 0.010
**10g**	0.004 ± 0.009	0.004 ± 0.010
**10h**	0.034 ± 0.012	0.008 ± 0.019
**10i**	0.003 ± 0.009	0.021 ± 0.010
**10j**	0.035 ± 0.015	0.024 ± 0.010
**11a**	0.007 ± 0.010	0.011 ± 0.008
**11b**	0.018 ± 0.012	0.030 ± 0.011
**11c**	0.045 ± 0.017	0.037 ± 0.012
**11d**	0.008 ± 0.010	0.014 ± 0.008
**12**	0.015 ± 0.013	-0.007 ± 0.010
**13**	0.004 ± 0.009	0.014 ± 0.008
**14**	-0.011 ± 0.007	0.001 ± 0.006
**15**	0.036 ± 0.018	-0.006 ± 0.008
**16**	0.007 ± 0.010	0.015 ± 0.009
**17**	0.001 ± 0.009	0.003 ± 0.007
**18**	0.002 ± 0.012	0.000 ± 0.009
**19**	0.004 ± 0.009	0.003 ± 0.007
**20**	-0.003 ± 0.008	-0.006 ± 0.005
**21**	0.069 ± 0.024	-0.017 ± 0.006
**22**	0.321 ± 0.085	-0.021 ± 0.006

Table S3. Lipidation of Compound 8. Extracted ion chromatograms are of the calculated *m*/*z* ± 0.005 of the $[M+H]^+$ ion for the lipidated compound.

<table>
<tr><td></td><td colspan="2">Extracted Ion Chromatograms</td></tr>
<tr><td></td><td>24 h</td><td>72 h</td></tr>
<tr><td>DOPC</td><td>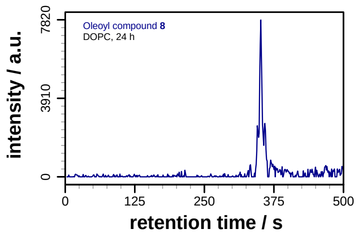
</td><td>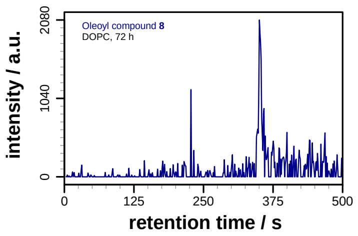
</td></tr>
<tr><td>POPC</td><td>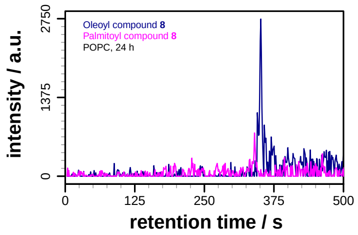
</td><td>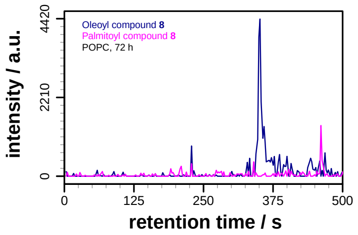
</td></tr>
<tr><td>OPPC</td><td>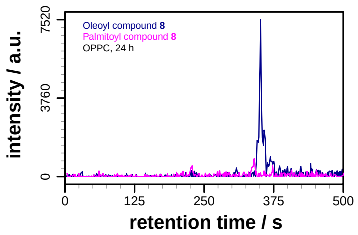
</td><td>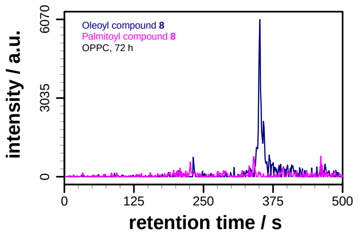
</td></tr>
<tr><td>DOPC/DOPS</td><td>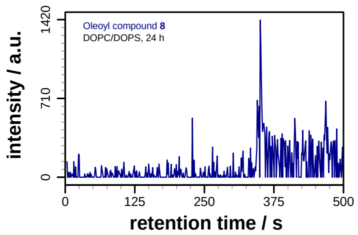
</td><td>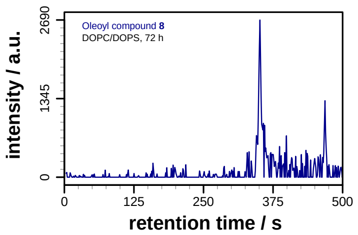
</td></tr>
<tr><td></td><td colspan="2">Representative Spectra</td></tr>
<tr><td></td><td>MS</td><td>MS/MS</td></tr>
<tr><td>Oleoyl</td><td>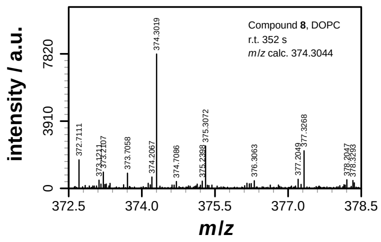
</td><td>-</td></tr>
<tr><td>Palmitoyl</td><td>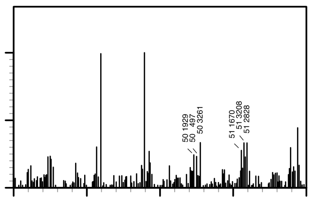</td><td>-</td></tr>
</table>

Table S4. Lipidation of Compound 9a. Extracted ion chromatograms are of the calculated *m*/*z* ± 0.005 of the $[M+H]^+$ ion for the lipidated compound.

	Extracted Ion Chromatograms 24 h	*Extracted Ion Chromatograms* 72 h
DOPC	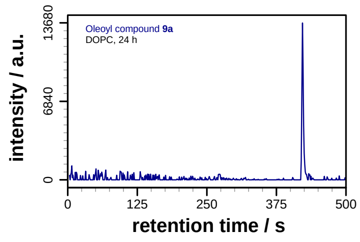	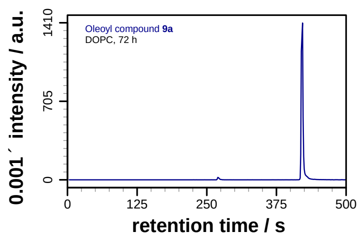
POPC	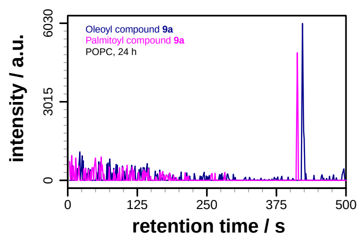	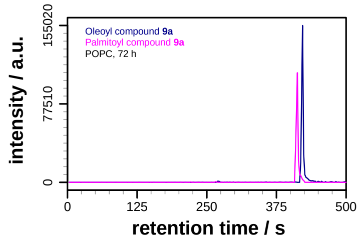
OPPC	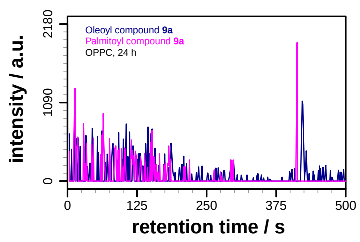	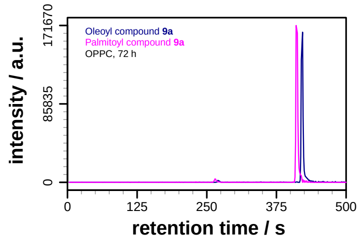
DOPC/DOPS	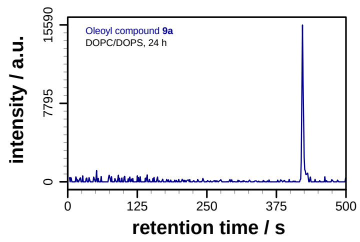	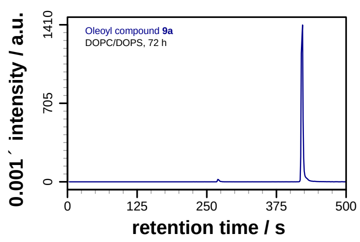
	Representative Spectra	
	MS	MS/MS
Oleoyl	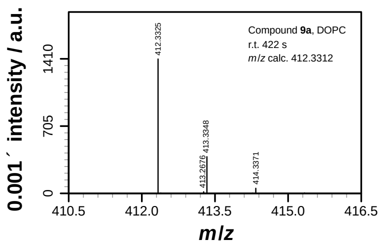	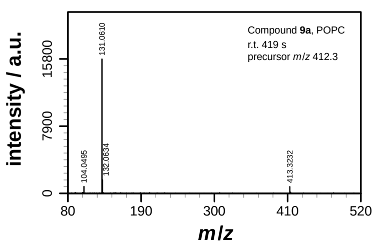
Palmitoyl	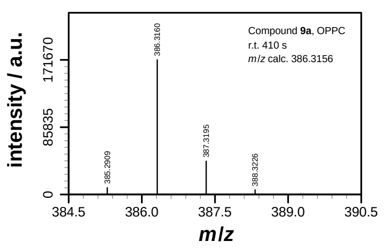	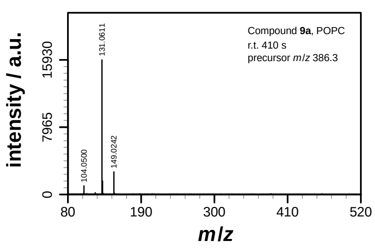

Table S5. Lipidation of Compound 10a. Extracted ion chromatograms are of the calculated *m*/*z* ± 0.005 of the $[M+H]^+$ ion for the lipidated compound.

	Extracted Ion Chromatograms 24 h	72 h
DOPC	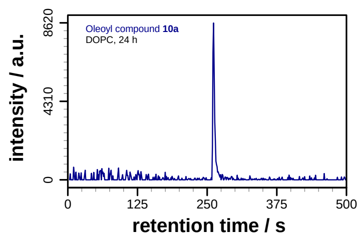	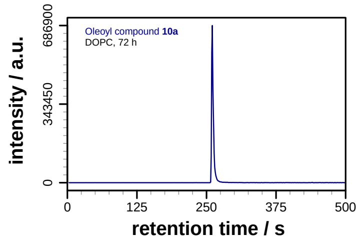
POPC	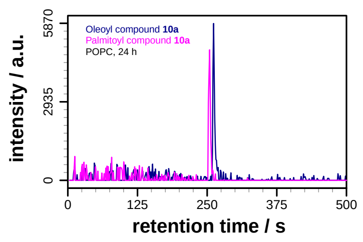	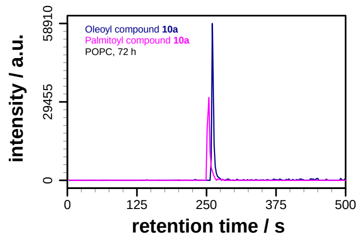
OPPC	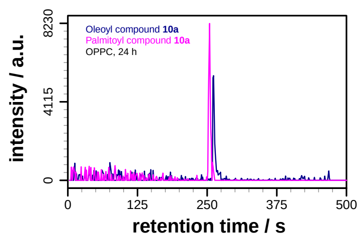	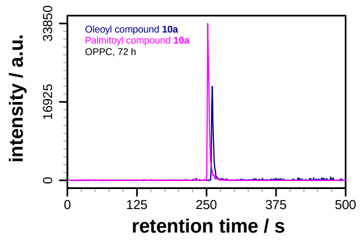
DOPC/DOPS	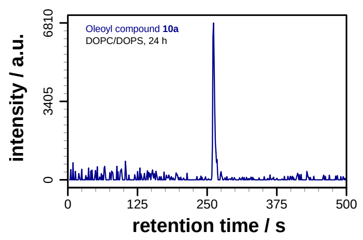	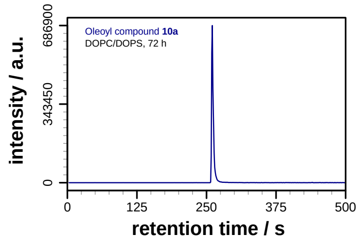
	Representative Spectra	
	MS	MS/MS
Oleoyl	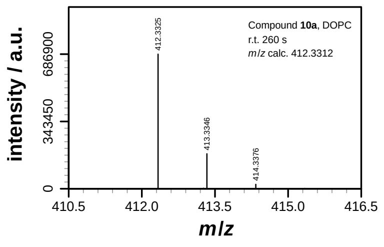	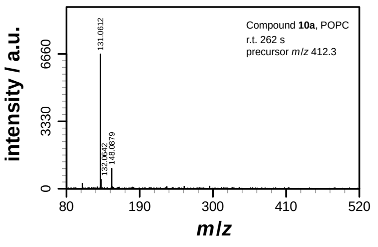
Palmitoyl	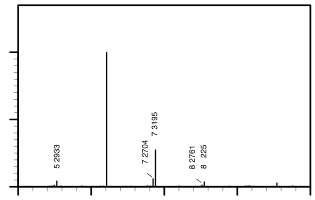	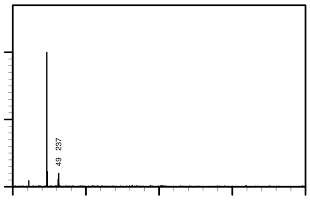

Table S6. Lipidation of Compound 10h. Extracted ion chromatograms are of the calculated *m*/*z* ± 0.005 of the $[M+H]^+$ ion for the lipidated compound.

	Extracted Ion Chromatograms 24 h	72 h
DOPC	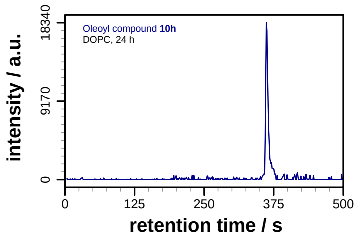	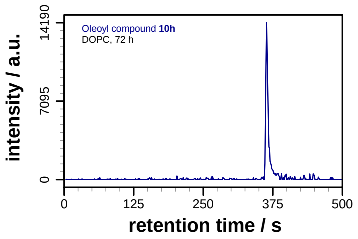
POPC	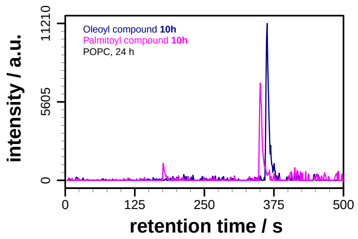	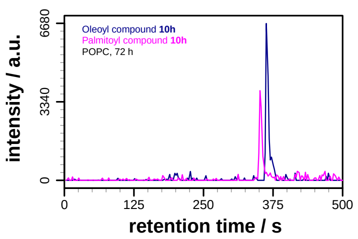
OPPC	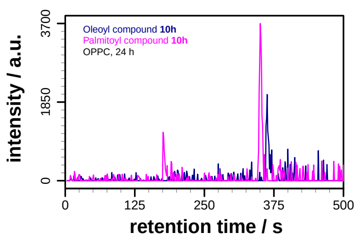	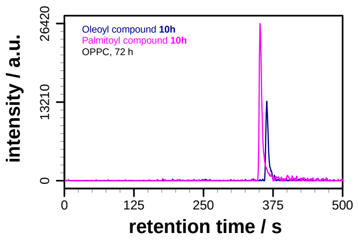
DOPC/DOPS	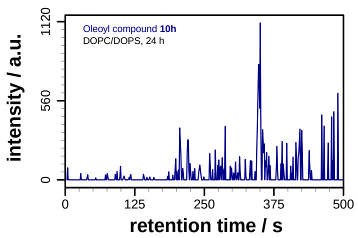	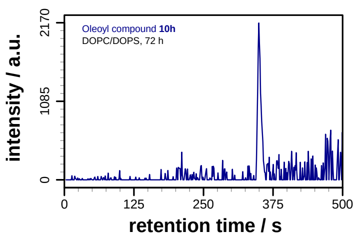
	Representative Spectra	
	MS	MS/MS
Oleoyl	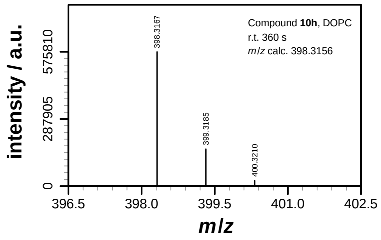	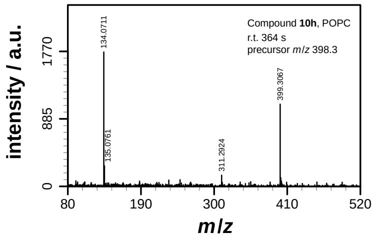
Palmitoyl	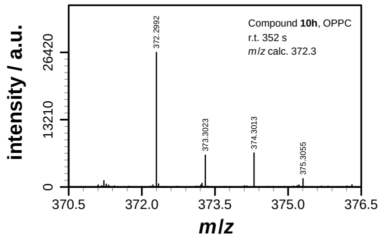	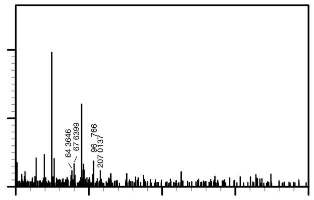

Table S7. Lipidation of Compound 10i. Extracted ion chromatograms are of the calculated *m*/*z* ± 0.005 of the $[M+H]^+$ ion for the lipidated compound.

Extracted Ion Chromatograms

	24 h	72 h
DOPC	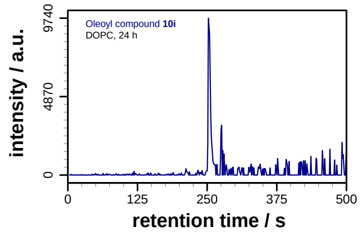	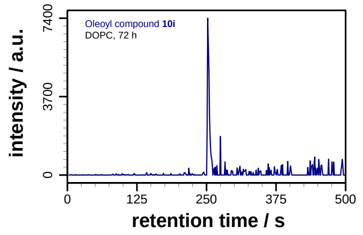
POPC	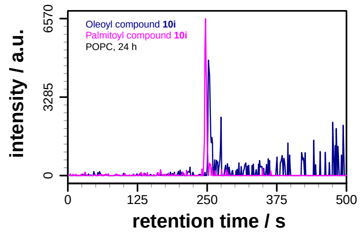	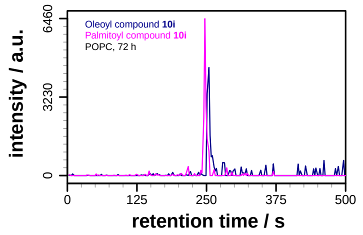
OPPC	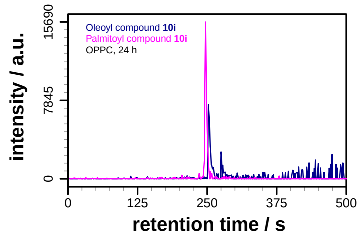	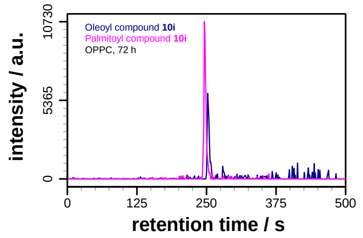
DOPC/DOPS	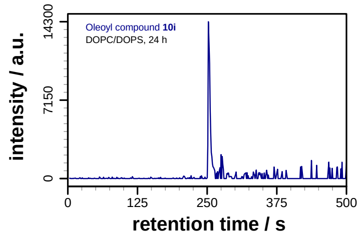	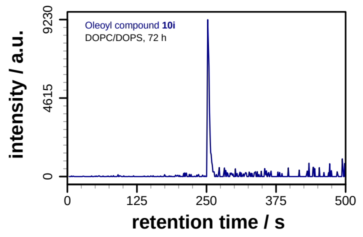
	Representative Spectra	
	MS	MS/MS
Oleoyl	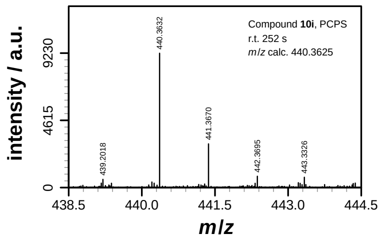	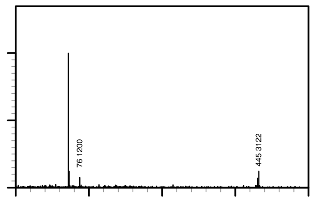
Palmitoyl	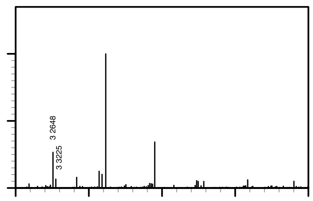	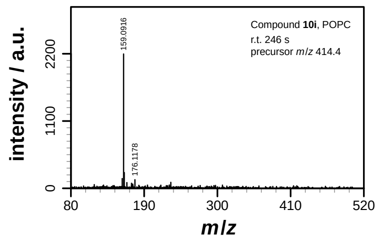

REFERENCES AND NOTES

1. S. Holzschuh, K. Kaeß, A. Fahr, C. Decker, Quantitative in vitro assessment of liposome stability and drug transfer employing asymmetrical flow field-flow fractionation (AF4). *Pharm. Res.* **33**, 842–855 (2016).
2. N. J. Zuidam, D. J. A. Crommelin, Chemical hydrolysis of phospholipids. *J. Pharm. Sci.* **84**, 1113–1119 (1995).
3. M. Grit, D. J. Crommelin, Chemical stability of liposomes: Implications for their physical stability. *Chem. Phys. Lipids* **64**, 3–18 (1993).
4. A. Catalá, Lipid peroxidation modifies the picture of membranes from the "Fluid Mosaic Model" to the "Lipid Whisker Model". *Biochimie* **94**, 101–109 (2012).
5. A. Reis, C. M. Spickett, Chemistry of phospholipid oxidation. *Biochim. Biophys. Acta* **1818**, 2374–2387 (2012).
6. C. M. Spickett, A. R. Pitt, Oxidative lipidomics coming of age: Advances in analysis of oxidized phospholipids in physiology and pathology. *Antioxid. Redox Signal.* **22**, 1646–1666 (2015).
7. S. J. Singer, G. L. Nicolson, The fluid mosaic model of the structure of cell membranes. *Science* **175**, 720–731 (1972).
8. R. H. Dods, J. A. Mosely, J. M. Sanderson, The innate reactivity of a membrane associated peptide towards lipids: Acyl transfer to melittin without enzyme catalysis. *Org. Biomol. Chem.* **10**, 5371–5378 (2012).
9. R. H. Dods, B. Bechinger, J. A. Mosely, J. M. Sanderson, Acyl transfer from membrane lipids to peptides is a generic process. *J. Mol. Biol.* **425**, 4379–4387 (2013).
10. H. M. Britt, J. A. Mosely, J. M. Sanderson, The influence of cholesterol on melittin lipidation in neutral membranes. *Phys. Chem. Chem. Phys.* **21**, 631–640 (2019).
11. C. J. Pridmore, J. A. Mosely, A. Rodger, J. M. Sanderson, Acyl transfer from phosphocholinelipids to melittin. *Chem. Commun.* **47**, 1422–1424 (2011).
12. V. S. Ismail, J. A. Mosely, A. Tapodi, R. A. Quinlan, J. M. Sanderson, The lipidation profile of aquaporin-0 correlates with the acyl composition of phosphoethanolamine lipids in lens membranes. *Biochim. Biophys. Acta* **1858**, 2763–2768 (2016).
13. H. M. Britt, C. A. García-Herrero, P. W. Denny, J. A. Mosely, J. M. Sanderson, Lytic reactions of drugs with lipid membranes. *Chem. Sci.* **10**, 674–680 (2019).

14. M. Baciu, S. C. Sebai, O. Ces, X. Mulet, J. A. Clarke, G. C. Shearman, R. V. Law, R. H. Templer, C. Plisson, C. A. Parker, A. Gee, Degradative transport of cationic amphiphilic drugs across phospholipid bilayers. *Philos. Trans. A Math. Phys. Eng. Sci.* **364**, 2597–2614 (2006).
15. D. Casey, K. Charalambous, A. Gee, R. V. Law, O. Ces, Amphiphilic drug interactions with model cellular membranes are influenced by lipid chain-melting temperature. *J. R. Soc. Interface* **11**, 20131062 (2014).
16. H. Jespersen, J. H. Andersen, H. J. Ditzel, O. G. Mouritsen, Lipids, curvature stress, and the action of lipid prodrugs: Free fatty acids and lysolipid enhancement of drug transport across liposomal membranes. *Biochimie* **94**, 2–10 (2012).
17. A. Arouri, O. G. Mouritsen, Membrane-perturbing effect of fatty acids and lysolipids. *Prog. Lipid Res.* **52**, 130–140 (2013).
18. SciFinder, *Chemical Abstracts Service: Columbus, OH; Density; RN 50–52-2*; https://scifinder.cas.org [accessed August 2013 and May 2019]; calculated using ACD/Labs software, version 8.14; ACD/Labs 1994–2007.
19. S. G. Kimani, K. Geng, C. Kasikara, S. Kumar, G. Sriram, Y. Wu, R. B. Birge, Contribution of defective PS recognition and efferocytosis to chronic inflammation and autoimmunity. *Front. Immunol.* **5**, 566 (2014).
20. Y. Chai, K. Jiang, Y. Pan, Hydride transfer reactions via ion–neutral complex: Fragmentation of protonated N-benzylpiperidines and protonated N-benzylpiperazines in mass spectrometry. *J. Mass Spectrom.* **45**, 496–503 (2010).
21. T. Pluskal, S. Castillo, A. Villar-Briones, M. Orešič, MZmine 2: Modular framework for processing, visualizing, and analyzing mass spectrometry-based molecular profile data. *BMC Bioinformatics* **11**, 395 (2010).
22. C. A. Smith, E. J. Want, G. O'Maille, R. Abagyan, G. Siuzdak, XCMS: Processing mass spectrometry data for metabolite profiling using nonlinear peak alignment, matching, and identification. *Anal. Chem.* **78**, 779–787 (2006).
23. R Core Team, *R: A Language and Environment for Statistical Computing* (R Foundation for Statistical Computing, Vienna, 2017); https://www.R-project.org.
24. N. Dyson, *Measurements and Models* (RSC, 1998), pp. 1–34.

25. S. A. Galal, A. S. Abdelsamie, H. Tokuda, N. Suzuki, A. Lida, M. M. ElHefnawi, R. A. Ramadan, M. H. E. Atta, H. I. El Diwani, Part I: Synthesis, cancer chemopreventive activity and molecular docking study of novel quinoxaline derivatives. *Eur. J. Med. Chem.* **46**, 327–340 (2011).
26. F. A. Alasmary, A. M. Snelling, M. E. Zain, A. M. Alafeefy, A. S. Awaad, N. Karodia, Synthesis and evaluation of selected benzimidazole derivatives as potential antimicrobial agents. *Molecules* **20**, 15206–15223 (2015).
27. J. A. Butera, W. Spinelli, V. Anantharaman, N. Marcopulos, R. W. Parsons, I. F. Moubarak, C. Cullinan, J. F. Bagli, Synthesis and selective class III antiarrhythmic activity of novel N-heteroaralkyl-substituted 1-(aryloxy)-2-propanolamine and related propylamine derivatives. *J. Med. Chem.* **34**, 3212–3228 (1991).
28. K. F. Ansari, C. Lal, D. L. Parmar, Some novel oxadiazolyl/azetidinyl benzimidazole derivatives: Synthesis and in vitro biological evaluation. *Synth. Commun.* **42**, 3553–3568 (2012).
